# Effects of exercise training on autonomic modulation and mood symptoms in patients with obstructive sleep apnea

**DOI:** 10.1590/1414-431X202010543

**Published:** 2021-03-15

**Authors:** C.E.L. Araújo, R. Ferreira-Silva, E.M. Gara, T.T. Goya, R.S. Guerra, L. Matheus, E. Toschi-Dias, A.G. Rodrigues, E.R.F. Barbosa, R. Fazan, G. Lorenzi-Filho, C.E. Negrão, L.M. Ueno-Pardi

**Affiliations:** 1 Instituto do Coração, Hospital das Clinicas, Faculdade de Medicina, Universidade de São Paulo, São Paulo, SP, Brasil; 2 Escola de Artes, Ciências e Humanidades, Universidade de São Paulo, São Paulo, SP, Brasil; 3 Departamento de Fisiologia, Faculdade de Medicina de Ribeirão Preto, Universidade de São Paulo, Ribeirão Preto, SP, Brasil; 4 Escola de Educação Física e Esportes, Universidade de São Paulo, São Paulo, SP, Brasil

**Keywords:** Exercise, Cardiac autonomic modulation, Spontaneous baroreflex sensitivity, Mood symptoms, Obstructive sleep apnea

## Abstract

We evaluated the effects of exercise training (ET) on the profile of mood states (POMS), heart rate variability, spontaneous baroreflex sensitivity (BRS), and sleep disturbance severity in patients with obstructive sleep apnea (OSA). Forty-four patients were randomized into 2 groups, 18 patients completed the untrained period and 16 patients completed the exercise training (ET). Beat-to-beat heart rate and blood pressure were simultaneously collected for 5 min at rest. Heart rate variability (RR interval) was assessed in time domain and frequency domain (FFT spectral analysis). BRS was analyzed with the sequence method, and POMS was analyzed across the 6 categories (tension, depression, hostility, vigor, fatigue, and confusion). ET consisted of 3 weekly sessions of aerobic exercise, local strengthening, and stretching exercises (72 sessions, achieved in 40±3.9 weeks). Baseline parameters were similar between groups. The comparisons between groups showed that the changes in apnea-hypopnea index, arousal index, and O_2_ desaturation in the exercise group were significantly greater than in the untrained group (P<0.05). The heart rate variability and BRS were significantly higher in the exercise group compared with the untrained group (P<0.05). ET increased peak oxygen uptake (P<0.05) and reduced POMS fatigue (P<0.05). A positive correlation (r=0.60, P<0.02) occurred between changes in the fatigue item and OSA severity. ET improved heart rate variability, BRS, fatigue, and sleep parameters in patients with OSA. These effects were associated with improved sleep parameters, fatigue, and cardiac autonomic modulation, with ET being a possible protective factor against the deleterious effects of hypoxia on these components in patients with OSA.

## Introduction

Obstructive sleep apnea (OSA) is characterized by recurrent decreases (hypopneas) or complete cessations (apneas) of airflow during sleep in the presence of a continued respiratory effort (obstructive airflow events) due to collapse of the upper airway, which leads to oxygen desaturations, arousals, and sleep fragmentation (1).

Repeated events of hypoxia and reoxygenation lead to an increase of reactive oxygen species (2), insulin resistance (3), systemic inflammation, vascular dysfunction, and increase in sympathetic nervous activity (4), placing OSA patients at risk for cardiovascular disorders and neuropsychological symptoms that include mood changes (5). These changes in patients with OSA lead to impaired daytime performance (6) and increased occupational and motor vehicle accidents (7), which translate into difficulties in social adjustment related to fatigue (8,9).

OSA patients have a cardiac autonomic dysfunction, including depressed baroreflex sensitivity (10,11), diminished vagal tone, and exaggerated cardiac sympathetic responsiveness (12,13). Previous studies also show that adults with mood disturbances experience reduced baroreflex sensitivity and autonomic modulation (14,15). Reduced heart rate variability (HRV) has been documented as a predictor of cardiovascular risk and mortality (16), suggesting that the presence of cardiac autonomic dysfunction has a significant impact on the prognosis of patients with OSA.

Accumulated evidence over the past decade shows that exercise training (ET) plays a role in OSA management. Several randomized controlled trials have demonstrated that ET reduces apnea-hypopnea index (AHI) (17,18) and improves objective and subjective sleep quality (17) independently of weight loss (17,18).

Additionally, chronic aerobic exercise can be an efficient treatment against mood disturbance compared with other interventions (19). Aerobic exercise combined with medication also improves mood disturbance, and this strategy is more efficient than medication alone (20). Few studies have investigated the effect of ET on the autonomic nervous system and mood status in patients with OSA. Therefore, the aim of this study was to investigate the effects of supervised ET on autonomic modulation and mood status in patients with OSA without other major comorbidities.

## Material and Methods

### Patients

Men and women aged 40 to 65 years who underwent conventional nocturnal polysomnography at the Sleep Laboratory of the Heart Institute, School of Medicine, University of São Paulo were selected for the present study. These individuals were part of a large study regarding ET on cerebral function in patients with OSA. Patients taking any medication, who had body mass index (BMI) ≥40 kg/m^2^, hypertension, cardiopulmonary disease, chronic kidney disease, diabetes mellitus, atrial fibrillation, arrhythmia, pacemaker, kidney failure, echocardiographic evidence of impaired left ventricular function (ejection fraction <45%), history of psychiatric disorders or other neurodegenerative disorders, smoking or alcohol abuse (2 or more drinks per day), any sleep apnea treatment, sleep disorders other than OSA, and circadian desynchrony (eg, shift workers) were excluded from the study.

Forty-four patients who were newly diagnosed with OSA (AHI >15 events per hour of sleep) were included in the study. They were randomly assigned on a 1:1 basis to the untrained group (n=22) and exercise-trained group (n=22). All patients were sedentary adults who had not exercised regularly for at least 3 months before enrolling in this study. All non-menopausal women were studied between the first and the fifth day after the onset of menstruation. All patients underwent echocardiographic evaluation at baseline. Full nocturnal polysomnography and maximal exercise capacity were performed at baseline and at the end of the study. The patients selected for the untrained group were asked to maintain their physical activity habits during the study. No patient in the untrained group reported changes in physical activity when questioned. This study was approved by the Research Committee of the Heart Institute (SDC 3536/10/125) and the Human Subject Protection Committee at the Clinical Hospital of the School of Medicine of the University of São Paulo (0833/10). The study is registered at Clinical Trials.gov (NIH: NCT002289625). All patients gave written informed consent to participate in the study. Full nocturnal polysomnography, echocardiographic measurements, maximal exercise capacity, cardiac autonomic evaluation, and a profile of mood states were performed at baseline and at the end of the study.

### Sleep analysis

OSA evaluation was performed according to the method previously described (21). All participants underwent a nocturnal polysomnography (Embla N7000, Medcare Flaga, Iceland). Monitoring included collection of electroencephalograms, electrooculograms, submental and tibial electromyography signals, airflow measurements (oronasal thermistor and pressure cannula), and movement of the chest and abdominal cords by bracing, measurement of oxyhemoglobin saturation by oximetry, and electrocardiography (ECG). Obstructive sleep apnea events were defined as 90% or greater reduction in respiratory amplitude for at least 10 s with thoracoabdominal effort. Sleep recordings were scored according to the criteria of Rechtschaffen and Kales (22). Hypopneas were defined as a 50% or greater reduction in respiratory range for at least 10 s associated with oxygen desaturation ≥3% and/or arousal. Individuals with >70% obstructive events were defined as individuals with OSA. The AHI was defined as the number of apnea and hypopnea events per hour of sleep. Sleep stages were classified according to the criteria of the American Academy of Sleep Medicine (23).

### Blood pressure

Clinical blood pressure (BP) readings were obtained from the left arm of subjects while seated, after 5 min of quiet rest, with a mercury sphygmomanometer. All subjects had at least 3 BP measurements obtained on separate occasions taken by one investigator. Systolic and diastolic BP was recorded at the first appearance and disappearance (phases I and V, respectively) of Korotkoff sounds. The subjects were classified as normotensive if the average systolic and diastolic BP levels were ≤140 or 90 mmHg, respectively.

### Profile of Mood States - POMS

The Profile of Mood States - POMS (24) scale has been one of the instruments most used to assess emotional and mood states as well as the variation of emotions associated with exercise and psychological wellbeing. The POMS version used in this research corresponded to a reduced version of the original scale. The validity design of this POMS version, as well as its internal consistency for Portuguese was published elsewhere (25). Briefly, the reduced version consists of a list of 42 adjectives for patients to describe themselves during the preceding week using a 5-point Likert scale format. Standard scoring of the POMS yields a global distress score referred to as total mood disturbance as well as scores for 6 subscales: Tension-Anxiety, Depression-Dejection, Anger-Hostility, Vigor-Activity, Fatigue-Inertia, and Confusion-Bewilderment.

### Cardiac function

Left ventricular function was assessed by an ultrasound device (Vivid E9, GE Healthcare, USA) using a 4-MHz multifrequency transducer. Left ventricular volumes and left ventricular ejection fraction were analyzed using 2-dimensional 2-chamber and 4-chamber examination by the Simpson's method. All study subjects underwent echocardiography performed by an echocardiographer blinded to the patient's history, who only was aware of the polysomnography before monitoring. All echocardiograms were performed, analyzed, and reported by the same echocardiographer, whose objective was to exclude the presence of structural heart disease.

### Cardiopulmonary exercise testing

Cardiorespiratory capacity was measured by a cardiopulmonary maximal exercise test on an electromagnetically braked cycle ergometer (Medifit 400L, Medical Fitness Equipment, The Netherlands), using a ramp protocol with workload increments of 10 or 15 W every minute at 60 rpm up to exhaustion. Oxygen uptake (VO_2_) and carbon dioxide production were determined by means of gas exchange on a breath-by-breath basis in a computerized system (SensorMedics, Vmax 229 model, USA). Anaerobic threshold was determined to occur at the breakpoint between the increase in the carbon dioxide production and VO_2_ (V-slope) or the point at which the end-tidal oxygen partial pressure curves reached its minimum value and began to rise. Respiratory compensation point was determined at the point at which the ventilatory equivalent for carbon dioxide was lowest before a systematic increase and when end-tidal carbon dioxide partial pressure reached a maximum and began to decrease. Peak VO_2_ was defined as the maximum attained VO_2_ at the end of the exercise period in which the subject could no longer maintain the cycle ergometer velocity at 60 rpm.

### Experimental protocol

The study was performed in a quiet, temperature-controlled room (21 to 22°C), in the morning at approximately the same time in the pre- and post-intervention with the subjects in the supine position. All signals were collected during 5 min of the subject being in the supine position with normal quiet breathing. Heart rate was continuously collected through lead II of an ECG recorded on a computer using the Windaq program (Dataq Insturments, USA) at a frequency of 1000 Hz. Simultaneously, arterial BP was monitored using a Finometer PRO^®^ (Finapress Medical System, The Netherlands), which provides noninvasive measurement on beat-to-beat basis. Respiratory rate was monitored using a piezoelectric thoracic belt (Pneumotrace II, model 1132, Respiration Transducer, UFI, USA) placed around the upper abdomen. All signals were recorded on a computer at a frequency of 1000 Hz and then analyzed in the Windaq program.

### Spontaneous baroreflex sensitivity analysis

For the analysis of baroreflex function and HRV, ECG and arterial pressure recordings were processed by customized computer software to generate series of successive values of RR-intervals (RR) and systolic arterial pressure (SAP).

Spontaneous baroreflex control (BRS) was analyzed by sequence method, as proposed by Bertinieri and co-workers (26). This method assumes that spontaneous arterial pressure ramps elicit baroreflex-mediated responses in RR length (27). Series of SAP were searched for increasing or decreasing ramps with at least 3 values of arterial pressure. When a pressure ramp (up or down) shows linear correlation (r>0.8) with RR changes at the same direction, a baroreflex sequence has been found. The spontaneous BRS is considered as the slope (ms/mmHg) of the linear regression between SAP and RR. The baroreflex gain of each subject is taken as the average slope, calculated from all the sequences.

### Heart rate variability analysis

Cardiac autonomic modulation was assessed from spontaneous fluctuations in the time series of RR. All the series were visually inspected, and artifacts or transients were manually removed. For time domain analysis we calculated the standard deviation of RR values (SDNN) and the root mean square of successive differences between normal heart beats (RMSSD). For frequency domain analysis of HRV, beat‐by‐beat series of RRs were converted to an evenly spaced series using cubic spline interpolation (10 Hz) and divided into half‐overlapping sequential sets of 512 data points (Welch periodogram). Segments with transients that could affect the calculation of power spectral density were excluded. A Hanning window was used to attenuate side effects, and the spectrum of each segment was calculated by fast Fourier transform (FFT). The spectra of RR were integrated and quantified in the very-low frequency (VLF: <0.04 Hz), low- frequency range (LF: 0.04 to <0.15 Hz), and in the high-frequency range (HF: 0.15 to 0.4 Hz). The absolute values of the RR interval spectral densities are reported in absolute values (ms^2^) and are also reported in normalized units (nu) as previously recommended for interpretation of spectral HRV indices in sleep research (28). The normalization procedure was performed by dividing the power of the LF or HF component by the total spectral power from which the power of the very low frequency (VLF) component had been subtracted. The LF/HF ratio was also calculated. Respiratory rate was measured in breaths per minute to certify that respiratory frequency of each subject was into the high-frequency range, i.e., between 0.15 and 0.4 Hz.

### Exercise training protocol

The supervised exercise training consisted of 72 exercise sessions at a frequency of 3 days per week. Each exercise session had a total duration of 60 min and consisted of 5 min of stretching the upper and lower limbs, 40 min of aerobic exercise on a cycle ergometer (in the first month for 30 min), 10 min of local strengthening exercises, and 5 min of muscle stretching (21). The intensity of aerobic exercise was determined by the corresponding heart rate of the anaerobic threshold and the respiratory compensation point (detected from the cardiorespiratory capacity test). Patients in the untrained group were told not to engage in systematic physical activity programs during the control period.

### Statistical analysis

The data are reported as means±SD (range) and proportions (gender). Unpaired Student's *t*-test was used to compare differences between groups (age, BMI, metabolic and functional parameters, baseline cardiovascular parameters, sleep parameters, POMS). Analysis of variance with repeated measures was used to compare within and between group differences in BMI, metabolic and functional parameters, cardiovascular parameters, sleep parameters, and POMS, before and after intervention. In the case of significance, *post-hoc* comparisons were performed with the Duncan test. P<0.05 was considered statistically significant. All analyses were performed using STATISTICA 12 software (StatSoft Inc., OK).

## Results

Ten patients were excluded from the study. In the untrained group, 2 patients were excluded from the analysis because they had artifacts in the collection signal, 1 patient started using the continuous positive airway pressure device (CPAP) during the study period, and 1 patient did not complete the exam protocols and was therefore excluded. In the exercise group, 1 patient was excluded because they had artifacts in the collection signal, 3 had inadequate breathing patterns for analysis, and 2 abandoned the physical training.

The baseline characteristics of the untrained and exercise-trained groups are shown in Table 1. No significant differences were observed between groups in age, weight, BMI, systolic BP, diastolic BP, and VO_2_ peak. There was also no significant difference in baseline sleep and autonomic modulation parameters between groups (P>0.05). Baseline POMS score was also similar between the groups regarding the 6 subscales (Table 2).

**Table 1:** Baseline physical characteristics, sleep parameters, and cardiac autonomic modulation of participants with obstructive sleep apnea in the untrained and exercise-trained groups.

Characteristics	Untrained(n=18)	Exercise-trained(n=16)	P
Gender, M/F	12/6	6/10	0.17
Age, years	54±8	50±6	0.20
Weight, kg	80±14	79±14	0.85
BMI, kg/m^2^	29±4	30±4	0.48
SBP, mmHg	123±11	129±15	0.90
DBP, mmHg	79±9	79±6	0.92
VO_2_ peak, mL·kg^-1^·min^-1^	26±6	22±5	0.10
Polysomnography			
AHI, events/h	45±27	44±31	0.91
O_2_ desaturation, events/h	34±27	38±31	0.69
Total arousal, events	216±121	195±114	0.45
Autonomic parameters			
RR, ms	942±129	891±135	0.26
SDNN, ms^2^	49±15	43±23	0.72
RMSSD, m^2^	40±19	31±23	0.23
BRS gain, ms/mmHg	10±4	8±5	0.45
LF, ms^2^	606±508	1161±2097	0.31
HF, ms^2^	658±717	711±1063	0.87
LF, nu	52±19	56±19	0.60
HF, nu	48±19	44±19	0.60
LF/HF	1.5±1	1.8±2	0.47

Data are reported as means±SD, except gender (n). There were no significant differences between the untrained and exercise-trained groups (*t*-test and chi-squared test (gender)). M: male; F: female; BMI: body mass index; SBP: systolic blood pressure; DBP: diastolic blood pressure; VO_2_ peak: oxygen uptake at peak exercise; AHI: apnea-hypopnea index; O_2_: oxygen; RR: ECG RR interval; SDNN: mean standard deviation of the RR intervals; RMSSD: root mean square of the differences between consecutive RR intervals; BRS: spontaneous baroreflex sensitivity; LF: low frequency band; HF: high frequency band; nu: normalized units.

**Table 2:** Baseline profile of mood states (POMS) subscales scores in patients with obstructive sleep apnea.

	Untrained (n=18)	Exercise-trained (n=16)	P
Tension	8±6	9±6	0.17
Depression	3±5	3±3	0.20
Hostility	4±5	4±4	0.85
Force	11±5	11±5	0.98
Fatigue	4±6	7±5	0.07
Confusion	5±2	5±2	0.92

Data are reported as means±SD. There were no significant differences between the untrained and exercise-trained groups (*t*-test).

### Effects of exercise training

ET attendance varied among patients. Seventy-two sessions or 100% of ET as planned was achieved in 40±3 weeks. The control group was paired with the ET group. ET and clinical follow-up caused no significant changes in body weight, BMI, and blood pressure (Table 3). After the intervention, a significant increase in VO_2_ peak was observed only in the ET group (P<0.05). A significant reduction in arousal was observed in the trained group (P<0.05). Comparisons between groups showed that the changes in AHI, O_2_ desaturation, and total arousals were significantly higher in the exercise group (P<0.05). The RR interval increased significantly after training. Gain of BRS was significantly higher in the exercise group compared with the untrained group (P<0.05). Furthermore, analysis of changes of RR, SDNN, RMSSD, and BRS were higher in the trained than in the untrained group (P<0.05).

**Table 3:** Effects of physical training on physical and physiological parameters of participants with obstructive sleep apnea.

	Untrained			Exercise-trained		
	Pre	Post	Delta	Pre	Post	Delta
Weight, kg	80±14	81±14	0.9±2	79±14	78±12	-1.0±4
BMI, kg/m^2^	29±4	29±4	0.1±1	30±4	30±4	-0.3±2
SBP, mmHg	123±11	123±15	-0.2±14	129±15	115±12	-8±13
DBP, mmHg	79±9	77±8	-2±13	79±6	77±6	-1±6
VO_2_ peak, mL·kg^-1^·min^-1^	26±6	25±7	-0.6±1	22±5	27±6*^†^	5±3^††^
Sleep parameters						
TTS, min	395±45	400±47	4±39	366±59	370±56	4±64
AHI, events/h	45±27	49±31	4±10	44±31	38±23*	-5±15^††^
O_2_ desat, events/h	34±27	43±30	9±20	38±31	35±22	-3±13^††^
Total arousal/events	216±121	222±128	6±40	195±114	155±69*^†^	-38±66^††^
Lowest O_2_ sat, %	82±7	81±6	-1±3	78±10	79±9	0.5±4
HRV parameters						
RR, ms	942±125	917±127	-32±85	891±135	932±113^†^	56±106^††^
SDNN, ms^2^	49±15	48±25	-1.6±20	43±23	50±22	6.9±16^††^
RMSSD, ms^2^	40±19	34±22	-6±20	31±23	36±22	5±13^††^
BRS gain, ms/mmHg	10±4	8±4	-2±4	8±5	10±6*^†^	2±3^††^
LF abs, ms^2^	606±508	915±1322	343±877	1161±2097	750±972	-410±168
HF abs, ms^2^	658±717	643±775	22±617	711±1063	669±715	-42±728
LF nu	52±19	57±14	8±18	56±19	54±22	-3±21
HF nu	48±19	43±15	-2±16	44±9	47±22	3±21
LF/HF	1.5±1	1.6±1	0.13±1	1.8±2	2.0±2	0.05±2

Data are reported as means±SD. *P<0.05, compared to untrained in post-intervention (between groups); ^†P<0.05 compared to pre-intervention (within group); ^††P<0.05 compared to the delta of the untrained group (repeated measures ANOVA). Pre: baseline measurement; Post: final measurement. SBP: systolic blood pressure; DBP: diastolic blood pressure; VO_2_ peak: oxygen uptake at peak exercise; TTS: total sleep time; AHI: apnea-hypopnea index; O_2_ desat: oxygen desaturation; O_2_ sat: oxygen saturation; HRV: heart rate variability; RR: ECG RR interval; SDNN: mean standard deviation of the RR intervals; RMSSD: root mean square of the differences between consecutive RR intervals; BRS: spontaneous baroreflex sensitivity; LF: low frequency in band; HF: high frequency band; nu: normalized units.^^

**Figure 1:**
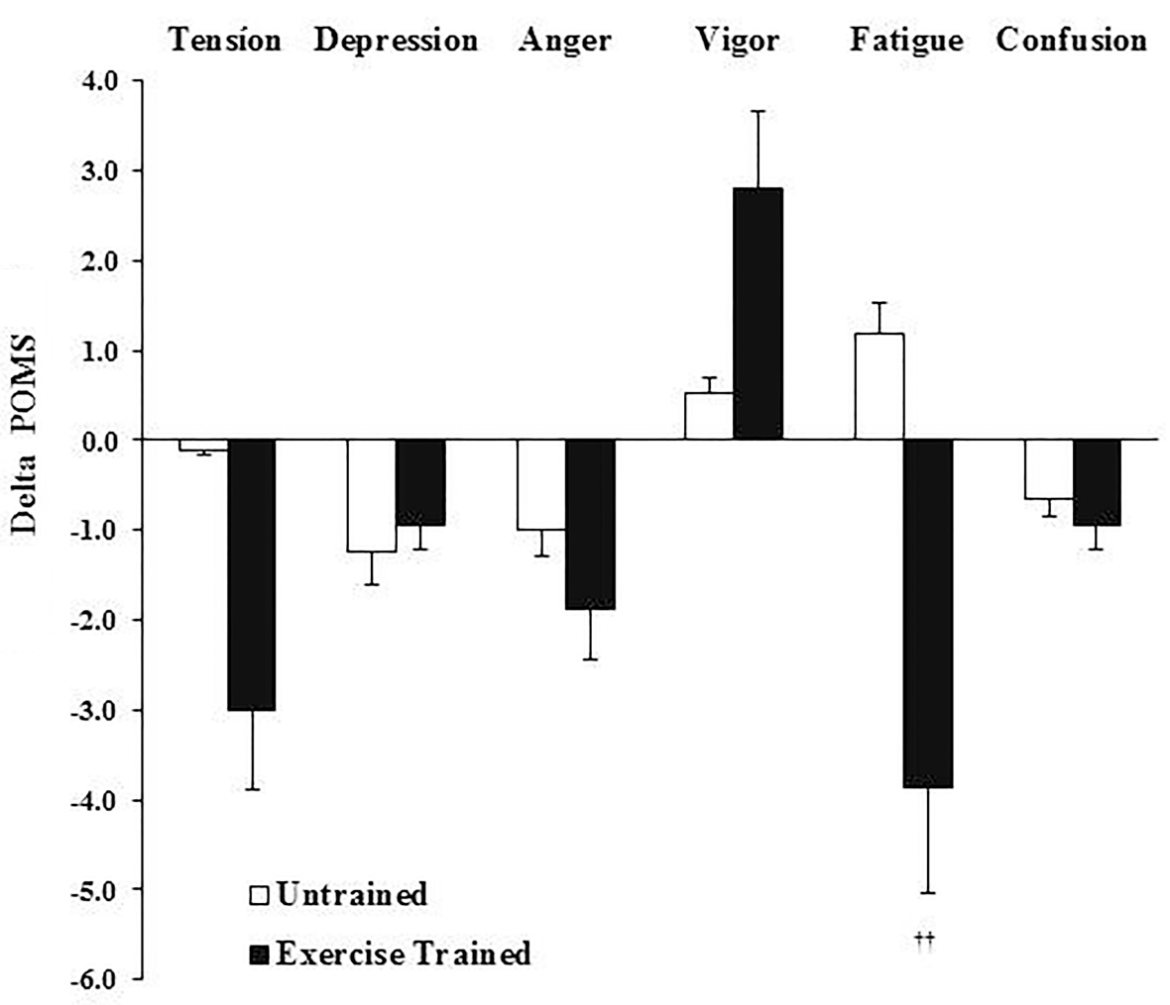
Delta of mood profile changes in patients with obstructive sleep apnea after intervention. Data are reported means±SD. ^††^P<0.05 *vs* Untrained (*t*-test between groups). POMS: Profile of Mood States.

Concerning the POMS scale, there was a significant reduction in fatigue (P<0.05) only in the exercise group (Table 4). Between-group comparisons showed the changes (Figure 1) in fatigue were significantly higher in the exercise group compared with untrained group (P<0.05). There was a positive correlation (r=0.60, P=0.02) between fatigue and AHI.

**Table 4:** Effects of physical training on the profile of mood state score in patients with moderate to severe sleep apnea.

	Untrained		Exercise-trained	
	Pre	Post	Pre	Post
Tension	8±6	7±4	9±6	6±6
Depression	3±5	2±2	3±3	2±3
Hostility	4±5	3±3	4±4	2±3
Force	11±5	12±5	11±5	14±5
Fatigue	4±6	6±5	7±5	3±4*†
Confusion	5±2	4±2	5±2	4±2

Data are reported as means±SD. *P<0.05, compared to untrained in post-intervention (between groups), ^†^P<0.05 compared to pre-intervention (within group) (repeated measures ANOVA). Pre: baseline measurement; Post: final measurement.

## Discussion

The main and new findings of the study are that ET in patients with OSA i) improved HRV and baroreflex function and ii) improved fatigue in the mood profile.

In previous studies, patients with OSA exhibited reduced baroreflex sensitivity and impaired autonomic modulation to the heart (15,29). The present study reveals that exercise training improved BRS in patients with OSA. Stiffening of large cardiothoracic arteries leads to a lower afferent trigger for a given change in BP, which reduces the afferent response capacity of the baroreceptors (30). Intermittent hypoxia and reoxygenation is a potential atherogenic factor, leading to damage of the wall of large arteries. A previous study found that middle-aged patients with severe OSA, without overt cardiovascular diseases, demonstrate early signs of atherosclerosis by means of increased arterial stiffness and carotid remodeling (31). ET may improve vascular structural change. Recently, Guerra et al. (21) showed that ET promoted a marked increase in forearm blood flow at rest and during handgrip exercise in patients with moderate to severe OSA (21).

It is possible that both mechanical and neural components play important roles in BRS regulation. The rapid heart period responses to baroreflex engagement in humans are predominantly mediated through efferent vagal activity (32). The present study shows that RR interval (index of vagal activity) was significantly increased by ET. The mechanisms involved in the improvement of HRV and BRS were not the scope of the present study. However, we can suggest that the improvement in BRS was due to an increased vascular structure, as well as endothelial factors (through the release of vasodilating substances). These responses ameliorate the vascular properties and, in consequence, facilitate the BRS (30,33).

Our results are in line with a previous study (34) that measured overnight HRV using a Holter ECG before and after 9 months of ET in patients with moderate OSA. The authors found that the RR interval increased in the exercise-trained group, whereas the control group had decreased parasympathetic and overall HRV parameters. It is possible that the effects of ET on autonomic modulation may extend even into the nocturnal period. In the present study, the improvement in cardiorespiratory capacity together with higher parasympathetic predominance may attenuate effects of hypoxia-related neural, vessel wall properties, and mechanical changes in the heart that lead to a decrease in BRS. The higher parasympathetic modulation in the exercise-trained group may also be a cardioprotective factor that contributes to the increasing of the fibrillation threshold as well as greater BP buffering action, including those caused by intermittent hypoxia.

A possible explanation for improving sleep parameters, more specifically those changes in AHI, arousal index, and O_2_ desaturation in the exercise-trained group, was due to the fact that ET would attenuate the narrowing or collapse of the upper airways by the redistribution of rostral fluid in the neck, reducing the severity of OSA (35). Alternatively, the increase in muscle tone of the genioglossus (36) reduces hypoxia and arousal episodes during sleep even without a significant change in body weight (21,35).

Fatigue is a common complaint in patients with OSA (37). A previous study found a wide range of fatigue in patients with OSA, including approximately 42% reporting a significant amount of fatigue (38). Fatigue is very prevalent in conditions associated with obesity, psychological distress, or in conditions of insufficient physical activity (39). Fatigue encompasses physical and psychological factors, and like pain, fatigue is a symptom that is almost always assessed by self-report. In this investigation, the fatigue-inertia category item of POMS included signs and symptoms of burnout, exhaustion, low energy level, and tiredness. ET significantly reduced the fatigue-inertia category item of the mood profile in patients with moderate to severe OSA. In the present study, the greater reduction in AHI and sleep fragmentation in the ET patients contributed to stabilization of the sleep structure, restoring correct levels of oxyhemoglobin in the blood and decreasing respiratory effort leading to a more restorative sleep that may affect the fatigue factor. The positive correlation (r=0.60, P=0.02) between the delta fatigue item and the delta AHI in the ET group strengthen this idea. These findings were consistent with a previous investigation in which improvement in fatigue after CPAP treatment was reported (40).

The improvement in OSA severity with ET might have contributed to the reduction in fatigue in patients with OSA and likely to the improvement in stressful daily routine. Because fatigue is also reported as a risk factor for injuries in work accidents (8) or risk of car accidents (7), the significant reduction in fatigue with ET can minimize the risk for these dangerous situations in these patients. Further studies on the mechanism of improvement in cardiac autonomic modulation and fatigue with ET in patients with OSA are needed.

Seventy-two ET sessions, conducted 3 times a week for 6 months as planned were achieved by some patients. In fact, 72 exercise sessions varied in frequency (1 to 3) over a period of 40±3 weeks, which occurs in real life during human intervention protocols. Despite the limitation, ET was able to significantly improve autonomic modulation of heart rate, baroreflex sensitivity, fatigue, and sleep parameters in patients suffering with OSA.

Among patients with OSA and no comorbidities, supervised ET induced adaptations in cardiac autonomic functions, baroreflex sensitivity, and symptoms of fatigue. These effects are associated with greater cardioprotection and improvement in well-being in patients with moderate to severe OSA.
